# Acupuncture for Fibromyalgia: An Open-Label Pragmatic Study on Effects on Disease Severity, Neuropathic Pain Features, and Pain Catastrophizing

**DOI:** 10.1155/2020/9869250

**Published:** 2020-02-25

**Authors:** Marco Di Carlo, Giacomo Beci, Fausto Salaffi

**Affiliations:** Rheumatological Clinic, Università Politecnica Delle Marche, Jesi, (Ancona), Italy

## Abstract

The treatment of fibromyalgia syndrome (FMS) is still far from being optimally coded, and pharmacological strategies are often unsatisfactory. Acupuncture plays a role among nonpharmacological intervention approaches; however, there is still no clarity as to when to integrate it into therapy. The objective of this study is to explore the role of acupuncture, in terms of efficacy on main disease severity measures and pain features, in patients with nonresponsive disease, defining nonresponsive FMS characterized by a revised Fibromyalgia Impact Questionnaire (FIQ-R) ≥39 and a Patient Health Questionnaire 15-item (PHQ15) ≥5 despite optimal drug therapy. Patients were treated with weekly sessions, for a total of eight acupuncture sessions. At the baseline and at the end of the treatment cycle, a comprehensive clinical evaluation was carried out to evaluate improvements in terms of disease severity and impact on neuropathic pain features (measured with the painDETECT questionnaire (PDQ)) and pain catastrophizing (measured with the Pain Catastrophizing Scale (PCS)). At the end of the eight-week treatment, patients experienced a significant improvement in all evaluated parameters (for FIQ-R, PDQ, and PHQ15 *p* < 0.0001, for PCS *p*=0.001). Of particular note is the effectiveness on manifestations that are difficult to treat such as neuropathic pain features and on negative psychological perceptions such as pain catastrophizing. It can be stated that acupuncture can be proposed also in phases of high severity of disease. Intervention with multimodal strategies, including acupuncture, could be of great benefit to patients.

## 1. Introduction

Fibromyalgia syndrome (FMS) still represents an “hard to treat” disease, and current treatment strategies leave a large number of unmet needs in patients. The pharmacological approach is still unsatisfactory considering that drugs currently available provide only modest benefits on pain, often with significant side effects, without having substantial efficacy on fatigue and quality of life compared to placebo [1].

In 2016, the European League against Rheumatism (EULAR) experts updated the recommendations on the management of FMS, addressing a “weak for” agreement for the use of acupuncture in FMS. On the other hand, nonpharmacological approaches are recommended as first-line therapy in patients with FMS [2].

To date, several studies have explored the effects of acupuncture, all showing good effects on pain and health in general in FMS patients [3–8].

Some authors have also demonstrated its effectiveness through randomized double-blind studies [7, 8]. Evidence that acupuncture works within the FMS is now available. While until a few years ago, the analgesic effect of acupuncture was considered indistinguishable from that of the bias of studies [9] and consequently acupuncture was not recommended in the treatment of FM, the most recent systematic reviews have reconsidered this position and have confirmed a favourable opinion of acupuncture for FM [10].

However, there is no clarity on when to recommend a course of treatment with acupuncture, as it is unlikely to be adopted as a continuous treatment. In particular, it is not clear to which kind of patients (i.e., patients not treated with drug treatment and patients refractory to drug treatment) and in which phase of disease severity should be proposed. To the best of our knowledge, to date, there have been no studies that have precisely identified these two standards. Among the various studies carried out, there are no well-defined inclusion criteria based on indices of severity of disease and unresponsiveness to drug therapy. In the available studies, inclusion criteria have been mainly based on the visual analogue scale (VAS) of pain [4], on the nonimprovement with the reference drug therapy [7], or even specific inclusion criteria based on indicators related to the disease are not specified [8].

The symptoms of FMS are increasingly considered as a continuum, and the importance of continuity of distress caused by symptoms is also important for diagnostic-classification purposes [11]. Patients generally come to the physician's attention when the symptoms (e.g., diffuse pain, fatigue, cognitive complaints, and sleep disturbance) are moderate or severe. Also, for FMS, in parallel with other rheumatic diseases, a “treat-to-target” (T2T) approach has recently been proposed, in order to reduce as much as possible the personal burden and the socioeconomic costs of the disease [12]. Being able to measure the severity of a disease through validated clinimetric indices is the prerequisite of the T2T strategy.

Today, there are widely validated clinical instruments available, and categories of disease severity have also been proposed for FMS, with cutoff points easily applicable to disease severity indices such as the revised Fibromyalgia Impact Questionnaire (FIQ-R). These categories can be used to classify patients in remission, mild, moderate, and high disease severity [13].

In the light of the aforementioned background, the aims of the present study were to explore the role of acupuncture, in terms of efficacy on main disease severity measures and pain features, in patients with nonresponsive disease, defining the latter on the basis of precise criteria listed below.

## 2. Materials and Methods

### 2.1. Patients and Inclusion Criteria

From January 2018 to June 2019, patients with FMS were enrolled in the outpatient clinic of a third-level rheumatology centre that represents the regional referral for this condition. Patients were enrolled consecutively, compatibly with their possibility of reaching the centre weekly for acupuncture sessions.

The diagnosis of FMS was made in all patients in accordance with the 2010 American College of Rheumatology criteria [11].

To define nonresponsive FMS, reference was made to the FIQ-R and to Patient Health Questionnaire 15-item (PHQ15) cutoffs proposed by Häuser and colleagues in 2017 [12]. These cutoffs considered the achievement of a FIQ-R <39 and a PHQ15 <5 to be a target for remission of somatic symptom burden. Therefore, patients not in remission (FIQ-R ≥39 and PHQ15 ≥5) were included in this study. In addition to the clinimetric definition, nonresponsiveness has also been defined at the pharmacological level. In particular, patients who have been on optimal and stable drug therapy for at least three months have been enrolled, defining as optimal and stable drug therapy the combination duloxetine 60 mg/day and pregabalin 300 mg/day. Patients who were not in remission and who were intolerant to the optimal and stable drug therapy (pregabalin duloxetine and/or pregabalin titrated at the respective dosages mentioned above) were included as long as the dosage of the drug therapy had been unchanged for at least three months. During the course of acupuncture treatment, patients were allowed to receive paracetamol (up to 3 grams/day) or tramadol (up to 150 mg/day) on demand.

All patients who had previously undergone acupuncture (even for indications other than FMS) were excluded from the scope of the study. Patients with concomitant life-threatening illnesses (e.g., heart failure or uncontrolled arrhythmias, severe chronic renal failure, liver failure, active neoplasms, and active infections) or with widespread skin diseases that contraindicated acupuncture were excluded. Patients with conditions that could mimic the symptoms of FMS or invalidate the disease assessment (e.g., chronic inflammatory joint diseases, polymyalgia rheumatica, hereditary myopathies or myositis, Lyme disease, hypermobility syndrome, hormonal imbalances caused by thyroid diseases or other uncontrolled endocrinological diseases, coeliac disease and chronic inflammatory bowel disease, multiple sclerosis, Parkinson's disease, peripheral neuropathies, posttraumatic stress disorder or major depression, and opioid-induced hyperalgesia) were also excluded [14].

All the procedures carried out in this study have been conducted in accordance with the 1964 Declaration of Helsinki. Patients signed informed consent to participate in the study, and the local ethics committee approved the protocol.

### 2.2. Acupuncture Treatment

The procedures carried out in this study are described according to the STandards for Reporting Interventions in Clinical Trials of Acupuncture (STRICTA) checklist [15]. In particular, all patients underwent a course of weekly whole-body acupuncture sessions for a total of eight consecutive weeks of treatment. Each session lasted 30 minutes, for a total dosage of 240 minutes of manual acupuncture per patient.

**Figure 1:**
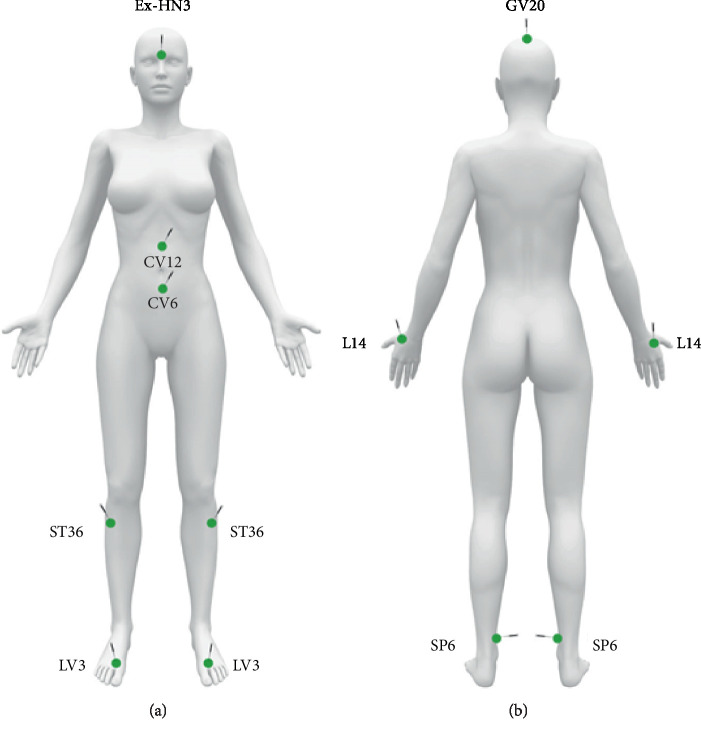
Manikin with the visual description of the location of acupuncture points used in this study.

All treatment sessions were conducted with needles with a guide tube, with the size of 0.25 × 25 mm (Huanqiu®), handled until the de Qi achievement. The following acupoints were used in all the patients for all the sessions: LV3, LI4, SP6, ST36, CV6, CV12, Ex-HN-3 (Yintang), and GV20 (Figure 1). Paired acupoints were treated bilaterally. Therefore, 12 needles per session were applied to each patient.

The acupoints were chosen in accordance with the principles of Traditional Cinese Medicine with the intention of moving *Qi* (LV3 + LI4), tonifying *Qi* and Blood (ST36 + SP6 + CV6 + CV12), raising *Qi* (GV20), and calming Shen (Ex-HN-3) [16].

All acupuncture sessions were performed by a physician (MDC) with nine years' experience in the field, with the certificate of acupuncture obtained at the end of a four-year course in 2014.

During the treatment sessions, the acupuncturist was not allowed to ask questions about the health status of the patients, and only minimal interaction with the patients was allowed. The acupuncturist was also blinded in relation to the precise number of sessions that were performed, and interaction with the patient was also prohibited in this sense. Throughout the study, the acupuncturist did not have access to the database and was blinded to the patients' clinimetric data.

No control group was used in this study, in particular, no control group based on sham acupuncture. An explanation of this choice is provided in the Section 4.

### 2.3. Clinimetric Assessment

The clinical evaluation was carried out at the beginning of the acupuncture treatment and at the end of the treatment, for each patient one week after the end of the treatment. The clinimetric assessment based on patient-reported outcomes (PROs) was conducted by a single physician (GB, experienced in administering questionnaires) in all patients and for all scheduled visits, while the tender point count (TPC), in the established visits, was conducted by a rheumatologist (FS) with more than thirty years of experience in the field of FMS, blind to the results of PROs.

For each patient, the severity of the disease was assessed through the FIQ-R, the severity of the somatic symptoms through the PHQ15. Other characteristics of painful symptoms were also investigated by assessing the neuropathic pain features, through the painDETECT questionnaire (PDQ), and the pain catastrophizing, through the Pain Catastrophizing Scale (PCS). The main characteristics of each instrument are briefly described below.

The FIQ-R consists of 21 numerical rating scales (NRS) (range 0–10, where 10 represents the worst condition for all NRS), and investigates function, overall impact and symptoms, three fundamental domains of health status in patients with FMS. The questions refer to the previous seven days, the final score goes from 0 to 100 and is the sum of the scores of the three domains: the algebraic sum of the 9 NRS in the function domain is divided by three, that of the 2 NRS in the overall impact domain is considered as it is, and that of the 10 NRS of the symptoms domain is divided by two [17]. Disease severity according to FIQ-R is defined by the following cutoffs: FIQ-R ≤30 indicates remission, FIQ-R >30 and ≤45 indicates mild severity, FIQ-R >46 and ≤65 indicates moderate severity, and FIQ-R >65 indicates high severity [13].

The PHQ15 is an easy-to-administer questionnaire focused mainly on somatic symptoms. The cutoffs distinguish low (below 5), medium (between 5 and 10), and high (above 15) severity of symptoms. The questionnaire proved to be a good predictor of the general state of health and healthcare use in somatic symptom disorders. It also demonstrated its validity in FMS [18].

The PDQ is a questionnaire completely self-administered, based solely on symptoms (no objective examination is required), which investigates the neuropathic features of painful symptoms. It has been available for over 10 years and has been studied for different conditions, including FMS [19, 20]. PDQ studies perceptions (allodynia, hyperalgesia, dysesthesia, and sudden pain) related to the neuropathic components of pain through seven 6-point scales (where 0 for “never,” and 5 for “very strong”). Neuropathic qualitative characteristics (such as burning, tingling or prickling, light touch pain, sudden, cold or hot pain attacks, numbness, and slight pressure triggering pain) are recalled in the areas of the body where the pain radiates (the irradiation of the pain to be indicated on a manikin is attributed 2 points). A question explores the temporal pattern of pain (score from −1 to 1 depending on the selected pattern). The final score has a range from -1 to 38. For scores ≤12, there is a low probability (<15%) of neuropathic pain, while for scores ≥19, there is a high probability (>90%) of neuropathic pain. Scores between 13 and 18 are defined as an ambiguous result [21].

To investigate the psychological aspects of painful symptoms, patients completed the PCS [22]. The PCS investigates three dimensions (helplessness, rumination, and magnification) related to the pain symptom. PCS consists of 13 statements (six for helplessness, five for rumination, and two for magnification), expressed with a negative connotation and related to specific thoughts and feelings. Patients are asked on a 5-point scale (from 0 for “never” to 4 for “always”) how often they recall a single thought. The total score goes from 0 to 52, with the possibility of counting the three subscales (0–24 helplessness, 0–20 rumination, and 0–8 magnification). The PCS has proved to be internally consistent both for the total score and for the three domains, also showing stability over time [23].

Finally, TPC, although no longer necessary in recent diagnostic criteria, is still the only objective measure of FMS, and it is still a common rheumatological clinical practice to perform it. The degree of tenderness represents a measure of the distress caused by FMS, and some authors consider TPC as the “sedimentation rate” of distress [24].

### 2.4. Statistical Analysis

The results are described as mean and standard deviation, with 95% confidence interval for the mean. The expected improvement at the end of the acupuncture treatment course, for all the parameters under study, was analyzed through the one-way analysis of variance (ANOVA), corrected for age and disease duration, with the adjustment of Holms-Bonferroni. For values <0.05, *p* were considered significant, and analyses were performed with SPSS for Mac, version 17.0.

## 3. Results

 The treatment course was started in 102 patients. Of these 102 patients, six did not complete the planned number of acupuncture sessions for two reasons. In detail, the reasons for the suspension were in two patients (a 22-year-old male and a 19-year-old female, respectively) a poor tolerance to needle insertion, so it was decided to discontinue treatment at the second session, while in four patients (all female), the sessions were stopped due to the impossibility of reaching the hospital weekly.

The treatment course and clinical evaluation was completed in 96 patients (94.1% of patients who started treatment), who tolerated the treatment well in the absence of significant adverse events (in eight patients was registered the presence of mild ecchymosis).

Of these 96 patients, 85 (88.5%) were female and 11 (11.5%) male. In this cohort of patients, the mean age (standard deviation (SD)) was 50.6 (12.3) years, with a duration of disease (SD) of 5.6 (6.2) years and body mass index of 26.2 (5.8) kg/m^2^.

Thirty-two (33.3%) of them were on duloxetine + pregabalin combination treatment, 22 (22.9%) were taking pregabalin in monotherapy, 13 (13.5%) were taking duloxetine in monotherapy, while 29 (30.2%) patients were not taking either of the two reference molecules.

Although the ambitious goal of reaching the target of a FIQ-R <39 and a PHQ15 <5 was achieved only in a minority of patients (4.2%), the course of eight weekly sessions of treatment with acupuncture resulted in a significant improvement of health status in many respects.  Table 1 shows the descriptive statistics, basal, and final, of the clinimetric indices used.  Table 2 illustrates the results of the one-way ANOVA corrected for age and for FMS duration.

Starting from the key clinimetric index in assessing the severity of the FMS, i.e., the FIQ-R, there has been a significant improvement (*p* < 0.0001) both in the overall score of the index and in its subscale symptoms, function, and overall impact.

An overall improvement in FIQ-R was recorded in 82 of 96 patients (85.4%), and in 39 patients (40.6%), the improvement was clinically significant (>30%) according to the definition proposed by Bennett et al. [25]. In 14 patients (14.6%), FIQ-R worsened at the end of the eight-week treatment period.

According to the definition of disease severity proposed by Salaffi and colleagues [13], a total of 49 (51.0%) patients were in a state of remission or mild severity (FIQ-R ≤45), while in 28 (29.2%) patients was documented a remission (FIQ-R ≤30).

An important and significant improvement was also recorded for somatic symptoms, measured with the PHQ15 (*p* < 0.0001). There was also a marked improvement in neuropatic-like features estimated by PDQ (*p* < 0.0001). If the pretreatment median values of PDQ started from 20, the median values of 15.5 were reached, below the cutoff threshold for the presence of neuropathic pain. The TPC also revealed a significant reduction in the number of tender points that could be detected on objective examination at the end of treatment (*p* < 0.0001).

Finally, at the end of the acupuncture treatment cycle, significant changes in the catastrophic attitude to pain in patients with FMS have been documented. In particular, the overall score of PCS improved significantly (*p*=0.001), as did helplessness (*p*=0.001) and rumination (*p*=0.003). For the magnification was recorded a smaller improvement (*p*=0.129).

## 4. Discussion

This study showed how effectively an eight-week course of acupuncture treatment is effective in patients with severe FMS. To the best of our knowledge, this is the first study to use well-defined inclusion criteria, based not only on the symptom pain but on precise clinical criteria and criteria of drug treatment (nonresponse or intolerance to drug therapy with duloxetine or pregabalin). We believe that this approach can be a great help mainly for patients, but also for clinicians, providing them with a proposal on the modalities of prescription of acupuncture.

The effectiveness of acupuncture in FMS is well known [3–8]. An interesting study published in 2008 and conducted by Brazilian researchers randomized 58 female patients to 20 acupuncture sessions in addition to exercise and tricyclic antidepressants versus exercise and tricyclic antidepressants. At the end of the 20 sessions, there was a marked improvement in the painful symptoms, in the number of tender points, and in the scales of the Short Form-36 which, however, was lost over the months (the follow-up lasted a total of 24 months). For the first time, the addition of acupuncture to the usual care was considered [26]. In this work, the inclusion criteria were the presence of a VAS 4 and the use of antidepressants. Considering only the pain scale probably does not fully reflect the many facets of FMS.

Other more recent research has also used pain VAS (in this case, more than 5 and referring to the last week) as the only criterion for inclusion. In our opinion, the only assessment of pain remains a limit in assessing the severity of FMS. However, this study also confirms the effectiveness of 20 acupuncture sessions conducted in accordance with the diagnosis in TCM [4].

In a recent randomized trail conducted in the setting of primary care, it was shown that true personalized acupuncture on TCM diagnosis, compared to sham acupuncture, leads to a marked improvement in painful symptoms, also improving functional capacity and quality of life. In our opinion, again, a limit is represented by generic inclusion criteria and covering a wide range of symptoms, so it was enough to be affected by FMS in accordance with the criteria ACR 1990 [8].

In our work, we considered it appropriate to use stringent inclusion criteria to define a severe and refractory disease, based on the reference clinimetry. FMS is a symptomatological continuum, of which pain is a substantial part but which may not be the major part of the problems [11, 27].

In this study, we also demonstrated the positive effect of acupuncture on pain catastrophizing. Acupuncture, therefore, allows patients to distract themselves, at least temporarily, from the pervasiveness of the symptoms. In FMS, PCS has been shown to correlate with kinesiophobia [28]. Intervening with a treatment that improves pain catastrophizing could, therefore, establish a virtuous circle whereby patients would more easily be engaged in a route of physical exercise (“strong for” agreement in EULAR 2016 guidelines for exercise). PCS correlate also with anxiety and depression [28]. In an elegant study that used functional magnetic resonance imaging, Ellingson and coworkers demonstrated that the tendency to catastrophism interferes with neural processes involved in pain modulation in FMS [29]. Acupuncture can, therefore, have powerful central effects in pain modulation, potentially also affecting cognitive aspects.

High PDQ scores are frequent in patients with FMS, and in these patients, high PDQ is probably a measure of pain centralization rather than an expression of injury to the somatosensory system [30–32].

In any case, neuropathic pain features are classically difficult manifestations to treat, both pharmacologically and with acupuncture [33]. This study has shown that through acupuncture, sensations that evoke the presence of neuropathic pain undergo a significant improvement. The finding confirms the significant effect of acupuncture on centrally mediated symptoms.

To date, there was no data in the literature to provide the efficacy of acupuncture on pain castrophizing and neuropathic pain features in patients with FMS.

The potential limitations of the study should also be mentioned. First of all, this research can be criticized for the fact that no control group was conducted for sham acupuncture to distinguish the placebo components from the verum ones of the treatment. However, on the basis of what has already been observed by some researchers, the use of a sham acupuncture (both minimally invasive and in points outside the meridian), especially in the field of painful diseases, instead of reducing the bias probably introduces others since it is a sensory stimulation not inert [34, 35].

A second and a third limit are represented by the single centre recruitment and that all acupuncture sessions were performed by a single practitioner, so our study methods (inclusion criteria and treatment scheme) need to be evaluated on a larger scale. A fourth potential limit was to use a “prescription” acupuncture scheme that was the same for all patients. However, this allowed us to use the same “dose” of acupuncture in all participants, ensuring uniformity of treatment.

## 5. Conclusions

In this study, we demonstrated the short-term efficacy of an eight-week course of acupuncture, added to the ongoing drug therapy, in patients affected by severe FMS. Based on strict inclusion criteria, acupuncture may also be proposed in phases of high severity of disease. For the first time, acupuncture has been shown to be effective on disease features such as pain catastrophizing and neuropathic pain features.

Earlier intervention with multimodal strategies, including acupuncture, could be of great benefit to patients. Future research should be directed at revealing predictors of the response to acupuncture treatment.

## Figures and Tables

**Table 1:** Descriptive statistics of the clinimetric indeces and suscales total at baseline (1) and at the end of the eight consecutive weeks of acupuncture treatment (2).

		Mean	Standard deviation	95% confidence interval for mean	
				Lower bound	Upper bound
FIQ-R total	1	60.79	19.41	56.85	64.72
	2	46.40	22.59	41.82	50.98

FIQ-R function	1	17.54	6.49	16.22	18.85
	2	14.66	10.67	12.49	16.82

FIQ-R overall impact	1	11.03	5.43	9.93	12.13
	2	7.60	5.54	6.48	8.73

FIQ-R symptoms	1	32.21	9.55	30.27	34.15
	2	24.94	10.66	22.78	27.10

PDQ	1	19.77	7.68	18.21	21.33
	2	15.27	7.39	13.77	16.77

PHQ15	1	14.58	5.41	13.49	15.68
	2	10.47	5.46	9.36	11.58

TPC	1	16.92	1.30	16.65	17.18
	2	9.46	4.32	8.58	10.33

PCS total	1	27.54	12.43	25.02	30.06
	2	22.11	10.85	19.92	24.31

Helplessness	1	12.07	6.14	10.83	13.32
	2	9.22	5.38	8.13	10.31

Rumination	1	12.08	5.03	11.06	13.10
	2	9.98	4.58	9.05	10.91

Magnification	1	3.39	2.14	2.95	3.82
	2	2.93	2.02	2.52	3.34

Abbreviations: FIQ-R = revised fibromyalgia impact questionnaire; PDQ = painDETECT questionnaire; PHQ15 = patient health questionnaire 15-item; TPC = tender point count; PCS = pain catastrophizing scale.

**Table 2:** One-way analysis of variance (ANOVA), corrected for age and disease duration, with the adjustment of Holms-Bonferroni, of the clinimetric indeces and suscales after the eight consecutive weeks of acupuncture treatment.

		Sum of squares	df	Mean square	F	Sig.
FIQ-R	Between groups	9931.69	1	9931.692	22.375	<0.0001
	Within groups	84334.47	190	443.866		
	Total	94266.17	191			

FIQ-R function	Between groups	398.18	1	398.189	5.099	0.025
	Within groups	14836.94	190	78.089		
	Total	15235.13	191			

FIQ-R overall impact	Between groups	563.75	1	563.755	18.694	<0.0001
	Within groups	5729.86	190	30.157		
	Total	6293.62	191			

FIQ-R symptoms	Between groups	2536.79	1	2536.794	24.744	<0.0001
	Within groups	19479.22	190	102.522		
	Total	22016.02	191			

PHQ15	Between groups	812.63	1	812.630	27.497	<0.0001
	Within groups	5615.24	190	29.554		
	Total	6427.87	191			

PDQ	Between groups	972.00	1	972.000	17.091	<0.0001
	Within groups	10805.91	190	56.873		
	Total	11777.91	191			

TPC	Between groups	2670.08	1	2670.083	262.156	<0.0001
	Within groups	1935.16	190	10.185		
	Total	4605.25	191			

PCS total	Between groups	1413.75	1	1413.755	10.380	0.001
	Within groups	25877.57	190	136.198		
	Total	27291.32	191			

Helplessness	Between groups	391.02	1	391.021	11.724	0.001
	Within groups	6336.89	190	33.352		
	Total	6727.91	191			

Rumination	Between groups	212.52	1	212.521	9.179	0.003
	Within groups	4399.29	190	23.154		
	Total	4611.81	191			

Magnification	Between groups	10.08	1	10.083	2.322	0.129
	Within groups	825.22	190	4.343		
	Total	835.31	191			

PDQ	Between groups	972.00	1	972.000	17.091	<0.0001
	Within groups	10805.91	190	56.873		
	Total	11777.91	191			

TPC	Between groups	2670.08	1	2670.083	262.156	<0.0001
	Within groups	1935.16	190	10.185		
	Total	4605.25	191			

Abbreviations: FIQ-R = revised fibromyalgia impact questionnaire; PDQ = painDETECT questionnaire; PHQ15 = patient health questionnaire 15-item; TPC = tender point count; PCS = pain catastrophizing scale.

## Data Availability

The data used to support the findings of this study are available from the corresponding author upon reasonable request.
